# Activation of Inward Rectifier K^+^ Channel 2.1 by PDGF-BB in Rat Vascular Smooth Muscle Cells through Protein Kinase A

**DOI:** 10.1155/2020/4370832

**Published:** 2020-05-01

**Authors:** Chengchun Tang, Dong Wang, Erfei Luo, Gaoliang Yan, Bo Liu, Jiantong Hou, Yong Qiao

**Affiliations:** Department of Cardiology, Zhongda Hospital, Medical School of Southeast University, DingjiaQiao No. 87, Hunan Road, Nanjing, 210009 Jiangsu, China

## Abstract

Platelet-derived growth factor-BB (PDGF-BB) can induce the proliferation, migration, and phenotypic modulation of vascular smooth muscle cells (VSMCs). We used patch clamp methods to study the effects of PDGF-BB on inward rectifier K^+^ channel 2.1 (Kir2.1) channels in rat thoracic aorta VSMCs (RASMCs). PDGF-BB (25 ng/mL) increased Kir2.x currents (−11.81 ± 2.47 pA/pF, *P* < 0.05 vs. CON, *n* = 10). Ba^2+^(50 *μ*M) decreased Kir2.x currents (−2.13 ± 0.23 pA/pF, *P* < 0.05 vs. CON, *n* = 10), which were promoted by PDGF-BB (−6.98 ± 1.03 pA/pF). PDGF-BB specifically activates Kir2.1 but not Kir2.2 and Kir2.3 channels in HEK-293 cells. The PDGF-BB-induced stimulation of Kir2.1 currents was blocked by the PDGF-BB receptor *β* (PDGF-BBR*β*) inhibitor AG1295 and was not affected by the PDGF-BBR*α* inhibitor AG1296. The PDGF-BB-induced stimulation of Kir2.1 currents was blocked by the protein kinase A inhibitor Rp-8-CPT-cAMPs; however, the antagonist of protein kinase B (GSK690693) had marginal effects on current activity. The PDGF-BB-induced stimulation of Kir2.1 currents was enhanced by forskolin, an adenylyl cyclase (AC) activator, and was blocked by the AC inhibitor SQ22536. We conclude that PDGF-BB increases Kir2.1 currents via PDGF-BBR*β* through activation of cAMP-PKA signaling in RASMCs.

## 1. Introduction

Platelet-derived growth factor-BB (PDGF-BB) is considered the major stimulant for vascular smooth muscle cell (VSMC) transition from a contractile state (also termed differentiated) to a synthetic state (also termed dedifferentiated) [1–3].VSMC phenotype switching plays a critical role in the pathophysiology of arterial remodeling in many vascular diseases including hypertension, atherosclerosis, and restenosis after angioplasty [4]. However, the molecular mechanisms underlying PDGF-BB-induced VSMC phenotype switching are not entirely clear.

PDGF-BB binds to PDGF receptor *β* and subsequently activates extracelluar signal-regulated kinase pathways to induce proliferation in human VSMCs [2]. Endothlin-1 inhibits Kir currents in rabbit coronary arterial smooth muscle cells (SMCs) through activation of protein kinase C [5]. Previous studies have demonstrated activation of cyclic adenosine monophosphate (cAMP)/protein kinase A- (PKA-) and phosphatidylinositol-3-kinase (PI3K)/protein kinase B- (PKB- or Akt-) related PDGF-BB-induced VSMC migration and proliferation [5–9].

Inward rectifier K^+^ channel 2.1 (Kir2.1), encoded by the KCNJ2 gene, is a member of the classic inwardly rectifying potassium channel family (Kir2.x). The channels of this family are constitutively active and exhibit strong inward rectification [10]. Kir2.1 plays biophysical roles in coronary, cerebral, and basilar arterial VSMCs with adenosine increasing Kir2.1 currents via the A3 receptor through activation of PKA in rabbit coronary arterial VSMCs [11–13]. In a previous study, we demonstrated that knockdown of Kir2.1 gene expression inhibits PDGF-BB-induced proliferation, migration, the rat VSMC phenotype, and postballoon injury intimal hyperplasia [14]. However, the detailed molecular mechanisms, particularly the electrophysiological regulation, have not been fully explored.

To address these questions, we studied the regulatory mechanisms of Kir2.1 by PDGF-BB in rat thoracic aorta VSMCs (RASMC) using the whole-cell patch clamp technique and Western blot analysis.

## 2. Materials and Methods

### 2.1. Animals and Ethical Considerations

Male Sprague-Dawley rats (150–180 g) were obtained from the Southeast University Animal Center. The animals were housed in a vivarium under controlled photocycle (12 h light/12 h dark) and temperature (22–25°C) conditions with free access to food and water. All procedures in the present study were conducted in accordance with the National Institutes of Health Guide for the Care and Use of Laboratory Animals and approved by the Care of Experimental Animals Committee of Southeast University (approval ID: SYXK-2015.4171).

### 2.2. Cell Preparation

The male Sprague-Dawley rats (150–180 g) were anesthetized with 10% chloral hydrate (3000 mg/kg, intraperitoneally) and exhibited no signs of peritonitis, pain, or discomfort following administration of 10% chloral hydrate. The Sprague-Dawley rats were anesthetized after 2-3 minutes; rats were euthanized with 6-9% isoflurane. If the Sprague-Dawley rats' hearts have not been beating, non-spontaneous breathing lasts 2-3 minutes, and there is no blinking reflex, the rats are considered dead. Rats were euthanized with an overdose of isoflurane, and RASMCs were isolated from the intimal-medial layers of the thoracic aorta as described [15]. Primary cells were cultured in Dulbecco's modified Eagle medium (DMEM)/F12 supplemented with 20% fetal bovine serum (FBS; Gibco BRL, Gaithersburg, MD) and 100 *μ*g/mL streptomycin-penicillin in an incubator at 37°C with 5% CO_2_. After passaging, RASMCs were cultured in DMEM/F12 with 10% FBS. Cells in the second to third passage were used for all experiments to prevent cell dedifferentiation.

### 2.3. Solutions

The bath solution (in mmol/L) was as follows: 3.5 KCl, 140 NaCl, 1.8 CaCl_2_, 1.5 MgSO_4_, 10 HEPES, and 10 glucose; the pH was adjusted to 7.4 with NaOH (1 mol/L). The pipette-filling solution (in mmol/L) was as follows: 40 KCl, 1.5 MgSO_4_, 5.0 KATP, 5.0 EGTA, K-aspirate 110, and 10 HEPES; the pH was adjusted to 7.4 with NaOH (1 mol/L). For single-channel recording, the extracellular pipette solution (in mmol/L) was as follows: 145 KCl, 10 HEPES, 10 glucose; the pH was adjusted to 7.4 with KOH (1 mol/L). The intracellular bath solution (in mmol/L) was as follows: 145 KCl, 1.2 MgCl_2_, 10 HEPES, 0.1 EGTA; the pH was adjusted to 7.38 with KOH (1 mol/L).

### 2.4. Drugs

All pharmacological compounds were prepared as aqueous or dimethyl sulfoxide stock solutions of >1,000 times the concentration used during the experiment. Recombinant mouse PDGF-BB was purchased from BioLegend (San Diego, CA, USA). AG1296, AG1295, GSK690693, and forskolin were purchased from Sigma (St. Louis, MO, USA). Rp-8-CPT-cAMPs and SQ22536 were purchased from BIOLOG Life Science Institute (La Jolla, CA, USA).

### 2.5. Cell Transfection of Kir2.x

Transfection was performed as previously described [5].

### 2.6. Reverse Transcription-Polymerase Chain Reaction

Total cellular RNA was isolated with a TRIzol reagent (Invitrogen, USA) and reverse-transcribed to cDNA using the SYBR® PrimeScript® RT-PCR Kit (Takara, Japan) at 37°C for 15 min. The gene expression was evaluated by SYBR® Premix Ex Taq™ (Takara, Japan). GAPDH was used as a housekeeping gene, in order to normalize the expression target gene. The thermal cycling conditions were as follows: 30 seconds at 95°C for predenaturation, 40 cycles for 15 seconds at 95°C for denaturation, 1 minute at 59°C for annealing, and 10 seconds at 72°C for elongation. At the end of each cycle, the fluorescence emitted by the SYBR Green I was measured. After the completion of the cycling process, samples were immediately subjected to a temperature ramp for melting curve analysis. The relative gene expression was analyzed by the 2^−*ΔΔ*Ct^ method. The primer sequences of KCNJ2, GAPDH, and the 3 Kir2 subunits were as follows: KCNJ2, forward: 5′-TGGATGCTGGTTATCTTCTGC-3′ and reverse: 5′-AGCCTATGGTTGTCTGGGTCT-3′; GAPDH, forward: 5′-AGAAGGCTGGGGCTCATTTG-3′ and reverse: 5′-AGGGGCCATCCACAGTCTTC-3′; Kir2.1, forward: 5′-CGGTGGATGCTGGTTATCTT-3′ and reverse: 5′-GAAAACAGCAATTGGGCATT-3′; Kir2.2, forward: 5′-CCAGTGCAACATTGAGTTCG-3′ and reverse: 5′-GCGATGACCCAGAAGATGAT-3′; Kir2.3, forward: 5′-CCATCATCATTGTCCACGAG-3′ and reverse: 5′-GAAGACCACAGGCTCAAAGC-3′.

### 2.7. Electrophysiology

Patch clamping was performed as previously described [16, 17].

### 2.8. Statistics

All experiments were repeated at least three times. The results are presented as the mean ± SEM. Statistical analyses were performed using one-way ANOVAs. LSD test was used for comparison between ≤3 sets of data as a post hoc test. Bonferroni test was used for comparison between >3 sets of data. The differences between two groups were considered statistically significant at *P* < 0.05. SPSS 19.0 statistical software (SPSS Inc., Chicago IL, USA) was used for data analyses.

## 3. Results

### 3.1. Effects of PDGF-BB on Kir2.x Currents

**Figure 1:**
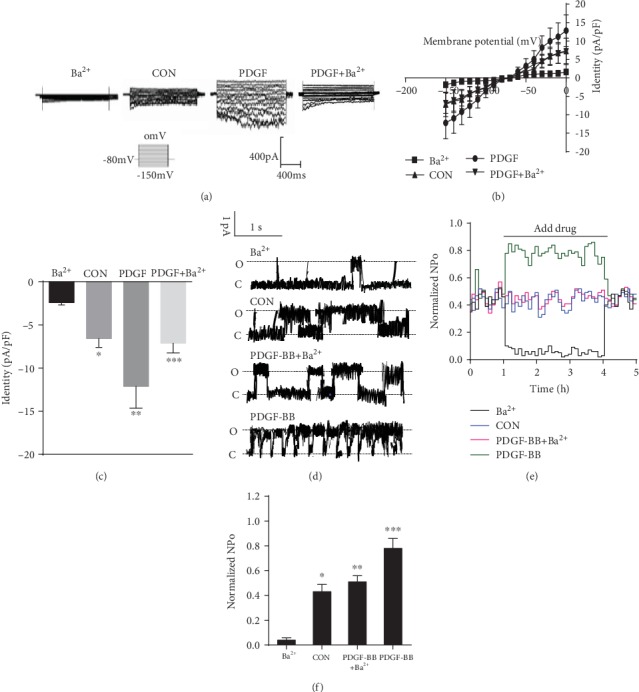
Effects of PDGF-BB on Kir2.x currents. (a) Original tracings illustrating currents determined in RASMCs. Membrane potential (E_m_) was held at -80 mV, and pulse potentials were applied from -150 mV to 0 mV. (b) Arithmetic mean ± SEM (*n* = 10) of current-voltage relationship curves. (c)Arithmetic mean ± SEM (*n* = 10) of the current amplitude at -150 mV of Kir2.x currents in Ba^2+^; CON. ^∗^*P* < 0.05 vs. Ba^2+^; PDGF-BB ^∗∗^*P* < 0.05 vs. CON; PDGF-BB+Ba^2+^. ^∗∗∗^*P* < 0.05 vs. PDGF-BB. (d) The “O” and “C” indicate the opened and closed states of Kir2.x currents, respectively. (e) Ba^2+^ attenuated and PDGF-BB increased the NPo of Kir2.x currents (at +70 mV) by 4.58 ± 0.33% and 78.82 ± 8.05% (^∗∗^*P* < 0.01 vs. CON, *n* = 5) compared to CON (43.29 ± 6.42%) (^∗^*P* < 0.01 vs. Ba^2+^, *n* = 5), and PDGF-BB+Ba^2+^ was 41.47 ± 6.13% (*P* < 0.01 vs. PDGF-BB, *n* = 5). (f) Time course of PDGF-BB action of Kir2.x currents. Averaged NPo plot (5 s bins) from five repeated perfusions of indicated compounds were normalized to control. CON means vascular smooth muscle cells without any drug intervention.

The membrane was clamped to 0 mV for 50 ms and then repolarized from +40 to -140 mV at a rate of 100 mV/s repeated once every second. For Kir recordings, the Na^+^ current was inactivated by holding at 0 mV, and the Ca^2+^ current was inhibited by adding 10 mM nitrendipine to the bath [18]. To exclude the involvement of ion channels outside the Ba^2+^-sensitive Kir2.x family, PDGF-BB was applied in the presence of Ba^2+^ (50 *μ*M). For Kir2.x recordings, the KATP current was inhibited by adding 100 nM glibenclamide to the bath. The fact that PDGF-BB could further weaken the inhibition of Ba^2+^ confirmed that PDGF-BB-target ion channels were Ba^2+^-sensitive Kir2.x [18]. Next, we investigated the effects of PDGF-BB on Kir2.x currents in RASMCs. We found that RASMCs employed Kir2.x currents, PDGF-BB (25 ng/mL) caused a significant increase, and 50 *μ*M Ba^2+^ significantly attenuated the current magnitude (Figure 1(a)). To confirm these data, the current-voltage relationship was measured from the voltage ramps (Figure 1(b)). The peak Kir2.x current densities of Ba^2+^, CON, PDGF-BB, and PDGF-BB+Ba^2+^ measured at -150 mV were −2.13 ± 0.23 pA/pF, −6.58 ± 0.93 pA/pF (*P* < 0.05 vs. Ba^2+^, *n* = 10), −13.58 ± 2.01 pA/pF (*P* < 0.05 vs. CON, *n* = 10), and −6.98 ± 1.03 pA/pF (*P* < 0.05 vs. PDGF-BB, *n* = 10), respectively (Figure 1(c)). To gain insight into the mechanism of PDGF-BB action on the Kir2.x channel, we tested the effects of PDGF-BB on the properties of a single Kir2.x channel expressed in RASMCs. A single-channel current was recorded with a pipette voltage of 70 mV using the excised inside-out configuration and then was identified by its unitary conductance (13–14.2 pS) and pharmacological sensitivity. The results showed that 50 *μ*M Ba^2+^ attenuated the average open probability (NPo) of Kir2.x, and PDGF-BB (25 ng/mL) significantly increased the NPo of Kir2.x. The NPo of the Kir2.x channel of Ba^2+^, CON, PDGF-BB, and PDGF-BB+Ba^2+^ measured at 4.58 ± 0.33%, 43.29 ± 6.42% (*P* < 0.01 vs. Ba^2+^, *n* = 5), 78.82 ± 8.05% (*P* < 0.01 vs. CON, *n* = 5), and 41.47 ± 6.13% (*P* < 0.01 vs. PDGF-BB, *n* = 5), respectively (Figure 1(d)). Common antagonists of Kir2.x including Ba^2+^ were applied to compare the effects of PDGF-BB on NPo of Kir2.x (Figure 1(e)). Furthermore, the time course of the PDGF-BB effect at a holding voltage of 70 mV was analyzed (Figure 1(f)).

### 3.2. PDGF-BB Activates Kir2.1 but Not Kir2.2 or Kir2.3 Channels in HEK293 Cells

**Figure 2:**
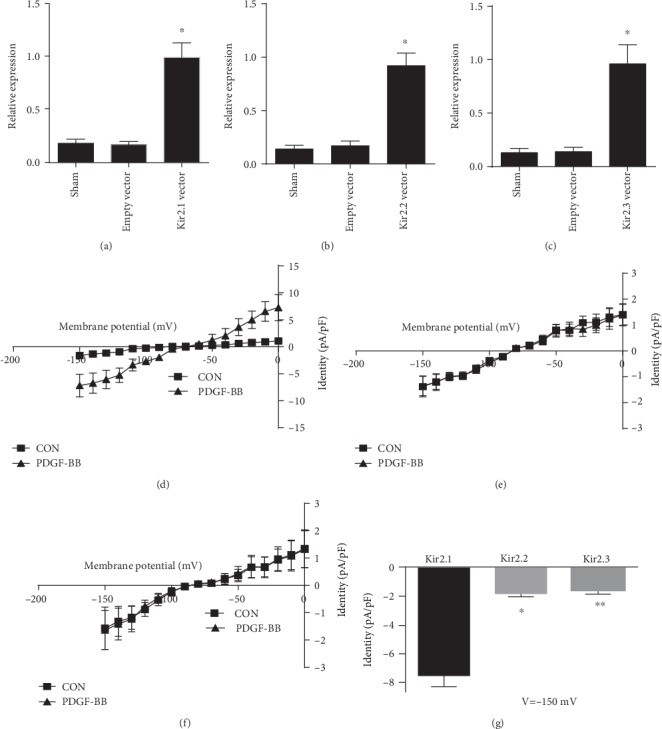
Effects of PDGF-BB on Kir2.1, Kir2.2, and Kir2.3 in transiently transfected HEK293 cells. (a–c) The transfections of the Kir2.1, Kir2.2, and Kir2.3 vector were verified by RT-PCR. (d–f) Arithmetic mean ± SEM (*n* = 10) of current-voltage relationship curves. (g) Arithmetic mean ± SEM (*n* = 10) of the current amplitude at -150 mV of Kir2.x currents in PDGF-BB; Kir2.1. ^∗^*P* < 0.05 vs. Kir2.2, Kir2.1; ^∗∗^*P* < 0.05 vs. Kir2.3. CON means vascular smooth muscle cells without any drug intervention.

First, we have verified that the transfections were successful by RT-PCR (Figures 2(a)–2(c)). To clarify the effects of PDGF-BB on rat Kir2.x, Kir2.1, Kir2.2, and Kir2.3, which constitute rat RASMC inward rectifier K^+^ channels (I_Kir_), they were individually expressed in HEK-293 cells. PDGF-BB remarkably increased rat Kir2.1 channels (46.43 ± 6.23% at -150 mV, *n* = 10) but not the currents of Kir2.2 (3.66 ± 1.54% at -150 mV, *n* = 10) or Kir2.3 (2.78 ± 1.03% at -150 mV, *n* = 10) (Figures 2(d)–2(g)). These results suggest that PDGF-BB only had significant agonist effects on Kir2.1 channels.

### 3.3. Effects of PDGF-BB*α* and PDGF-BB*β* Antagonists on PDGF-BB-Mediated Activation of Kir2.1 Currents

**Figure 3:**
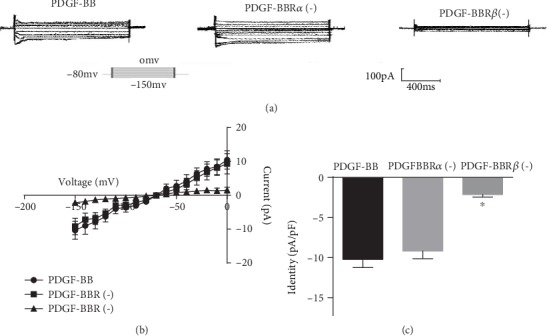
Effects of PDGF-BBR*α* (-) and PDGF-BBR*β* (-) on PDGF-BB-mediated activation of Kir2.1 currents. (a) Original tracings illustrating currents determined in RASMCs. E_m_ was held at -80 mV, and pulse potentials were applied from -150 mV to 0 mV. (b) Arithmetic mean ± SEM (*n* = 10) of current-voltage relationship curves. (c) Arithmetic mean ± SEM (*n* = 10) of the current amplitude at -150 mV of Kir2.1 currents in PDGF-BB; PDGF-BBR*α* (-). *P* > 0.05 vs. PDGF-BB; PDGF-BBR*β* (-). ^∗^*P* < 0.05 vs. PDGF-BB.

We tested the relative contributions of the PDGF-BB*α* and PDGF-BB*β* receptors (PDGF-BBR*α* and PDGF-BBR*β*) on PDGF-BB-mediated activation of Kir2.1 currents in RASMCs using the PDGF-BBR*α* effective inhibitor AG1296 (2 *μ*M) and the PDGF-BBR*β*-effective inhibitor AG1295 (2 *μ*M). PDGF-BBR*α* blockade (PDGF-BBR*α* (-)) had no effect on PDGF-BB-mediated Kir2.1 current activation, and the blockade of PDGF-BBR*β* (PDGF-BBR*β* (-)) significantly reduced the effects on PDGF-BB-mediated Kir2.1 current activity (Figure 3(a)). To confirm these data, the current-voltage relationship was measured from the voltage ramps (Figure 3(b)). The peak Kir2.1 current densities of PDGF-BB, PDGF-BBR*α* (-), and PDGF-BBR*β* (-) measured at -150 mV were −10.3 ± 1.66 pA/pF, −8.81 ± 1.83 pA/pF (*P* > 0.05 vs. PDGF-BB, *n* = 10), and −2.53 ± 0.52 pA/pF (*P* < 0.05 vs. PDGF-BB; *n* = 10), respectively (Figure 3(c)).

### 3.4. PKA and Akt Mediate the Inhibitory Effects of PDGF-BB on Kir2.1 Channels

**Figure 4:**
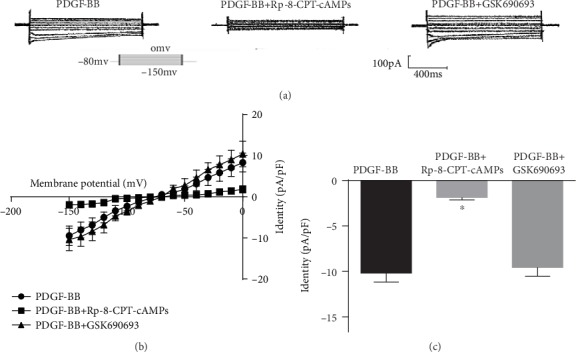
Effects of PKA and Akt on PDGF-BB-mediated activation of Kir2.1 currents. (a) Original tracings illustrating currents determined in RASMCs. E_m_ was held at -80 mV, and pulse potentials were applied from -150 mV to 0 mV. (b) Arithmetic mean ± SEM (*n* = 10) of current-voltage relationship curves. (c) Arithmetic mean ± SEM (*n* = 10) of the current amplitude at -150 mV of Kir2.1 currents in PDGF-BB; PDGF-BB+Rp-8-CPT-cAMPs. Rp-8-CPT-cAMPs (10 *μ*M). ^∗^*P* < 0.05 vs. PDGF-BB; PDGF-BB+GSK690693. GSK690693 (10 nM). *P* > 0.05 vs. PDGF-BB.

To test whether the activation of Kir2.1 currents by PDGF-BB was mediated by the cAMP/PKA and PI3K/Akt pathway, we determined the effects of specific PKA and Akt inhibitors (Rp-8-CPT-cAMPs and GSK690693, respectively) on the activation of Kir2.1 currents induced by PDGF-BB (25 ng/mL). The Rp-8-CPT-cAMPs (10 *μ*M) reduced the PDGF-BB-induced increase in the Kir2.1 currents to the control level in RASMCs (Figure 4(a)). GSK690693 (10 nM) had no significant effects on the PDGF-BB-induced activation of Kir2.1 currents in RASMCs (Figure 4(a)). To confirm these data, the current-voltage relationship was measured from the voltage ramps (Figure 4(b)). The peak Kir2.1 current densities of PDGF-BB, PDGF-BB+Rp-8-CPT-cAMPs, and PDGF-BB+GSK690693 measured at -150 mV were −8.05 ± 1.52 pA/pF, −2.42 ± 0.42 pA/pF (*P* < 0.05 vs. PDGF-BB, *n* = 10), and −10.36 ± 2.06 pA/pF (*P* > 0.05 vs. PDGF-BB, *n* = 10), respectively (Figure 4(c)).

### 3.5. Effect of Adenylyl Cyclase on PDGF-BB-Mediated Activation of Kir2.1 Currents

**Figure 5:**
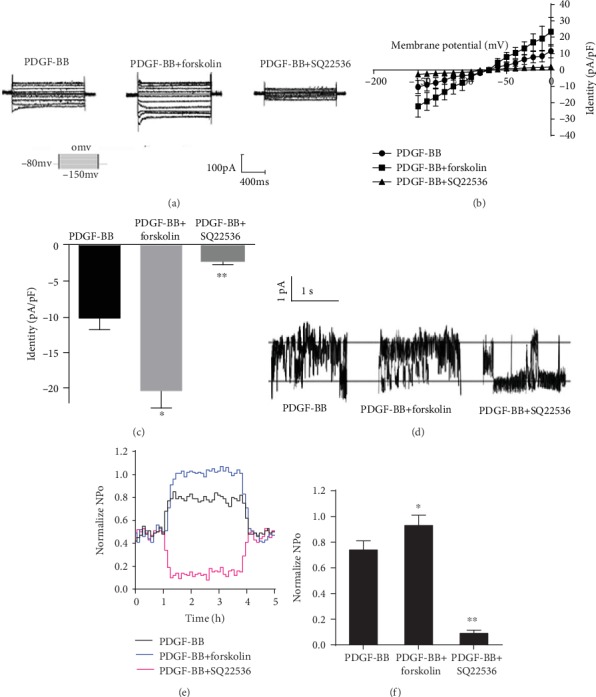
Effects of AC on PDGF-BB-mediated activation of Kir2.1 currents. (a) Original tracings illustrating currents determined in RASMCs. E_m_ was held at -80 mV, and pulse potentials were applied from -150 mV to 0 mV. (b) Arithmetic mean ± SEM (*n* = 10) of current-voltage relationship curves. (c) Arithmetic mean ± SEM (*n* = 10) of the current amplitude at -150 mV of Kir2.1 currents in PDGF-BB; PDGF-BB+forskolin. Forskolin (10 *μ*M). ^∗^*P* < 0.05 vs. PDGF-BB; PDGF-BB+SQ22536. SQ22536 (10 nM). ^∗∗^*P* < 0.05 vs. PDGF-BB. (d) The “O” and “C” indicate the opened and closed states of Kir2.1 currents, respectively. (e) The forskolin increased and SQ22536 decreased the NPo of Kir2.1 currents (at +70 mV) by 98.16 ± 8.61% (^∗^*P* < 0.05 vs. PDGF-BB, *n* = 5) and 8.73 ± 0.23% (^∗∗^*P* < 0.01 vs. PDGF-BB, *n* = 5) compared to PDGF-BB alone (70.23 ± 7.35%). (f) Time course of PDGF-BB action of Kir2.1 currents. Averaged NPo plot (5 s bins) from five repeated perfusions of indicated compounds was normalized to control.

As the effects of PKA on Kir2.1 channel currents may be produced either directly or by activation of adenylyl cyclase (AC), we added forskolin, an effective activator of AC, and SQ22536, an effective inhibitor of AC, to Kir2.1 currents induced by PDGF-BB (25 ng/mL). The results show that forskolin (10 *μ*M) significantly enhanced the PDGF-BB-induced increase in the Kir2.1 currents to the control level in RASMCs. The SQ22536 (10 nM) reduced the PDGF-BB-induced increase in the Kir2.1 currents to the control level in RASMCs (Figure 5(a)). To confirm these data, the current-voltage relationship was measured from the voltage ramps (Figure 5(b)). The peak Kir2.1 current densities of PDGF-BB, PDGF-BB+forskolin, and PDGF-BB+SQ22536 measured at -150 mV were −12.26 ± 2.67 pA/pF, −24.17 ± 4.65 pA/pF (*P* < 0.05 vs. PDGF-BB, *n* = 10), and −2.41 ± 1.01 pA/pF (*P* < 0.05 vs. PDGF-BB, *n* = 10), respectively (Figure 5(c)). To gain insight into the molecular mechanism governing PDGF-BB activation of the Kir2.1 channel, the single-channel currents were recorded with a pipette voltage of 70 mV using the excised inside-out configuration and then were identified by their unitary conductance (13–14.2 pS) and pharmacological sensitivity. The results showed that forskolin (10 *μ*M) increased and SQ22536 (10 nM) decreased the NPo of Kir2.1. The NPo of the Kir2.1 channel of PDGF-BB, PDGF-BB+forskolin, and PDGF-BB+SQ22536 were measured at 70.23 ± 7.35%, 98.16 ± 8.61% (*P* < 0.05 vs. PDGF-BB, *n* = 5), and 8.73 ± 0.23% (*P* < 0.01 vs. PDGF-BB, *n* = 5), respectively (Figure 5(d)). An AC activator and AC inhibitor were employed to compare the effects of PDGF-BB on NPo of Kir2.1 (Figure 5(e)). In addition, a time course of the drug effect at a holding voltage of 70 mV was analyzed (Figure 5(f)).

## 4. Discussion

Our observations revealed that PDGF-BB activated the Kir2.1 channel, the PDGF-BB-induced activation of the Kir2.1 channel was mediated by activation of the PDGF*β* receptor, and the response occurred via the activation of AC and PKA. Regulation of the proliferation, migration, and phenotypic modulation in VSMCs by PDGF-BB has been studied extensively [19]. Ion channel status is associated with several occlusive vascular diseases involving VSMC phenotypic modulation. For example, the switch toward intermediate-conductance Ca^2+^-activated K^+^ channel (BK_Ca_) expression may promote excessive neointimal VSMC proliferation, and dysfunction of K^+^ channels is linked to pulmonary arterial remodeling [20, 21]. Reduction in the L-type Ca^2+^ channel *α*1 subunit (Cav1.2) has been observed in rat aortic VSMCs during dedifferentiation and after balloon injury [22, 23]. Decreased K^+^ channel activity causes depolarization of the E_m_ and subsequently elevates free Ca^2+^ concentration in the cytoplasm via opening of Cav channels, which is required for VSMC proliferation and remodeling [21]. The phenotype-dependent plasticity of Kir channels may have relevance to vascular remodeling [24]. In VSMCs, only Kir2.1 has been identified [13]. Blood vessels in Kir2.2 knockout mice dilate normally in response to high K^+^ stimulation but not in vessels from Kir2.1 knockout mice [25]. Therefore, Kir2.1 seems to be a main subunit in the formation of classic Kir currents in these cells. Recently, we reported that PDGF-BB promotes expression of Kir2.1 channel protein in RASMCs. Electrophysiological studies demonstrated that the functional upregulation of BK_Ca_ is required for PDGF-BB-induced coronary SMC phenotypic modulation and migration [26]. In this study, we found that PDGF-BB increases Kir2.1 channel currents in RASMCs (Figure 2(d)). The single-channel results also revealed that PDGF-BB enhanced the NPo of the Kir2.1 channel (Figure 1(d)).

To date, four distinct types of K^+^ channels have been identified in VSMCs: voltage-gated K^+^ (Kv) channels, ATP-sensitive potassium (K_ATP_) channels, BK_Ca_ channels, and Kir channels [14]. Steady-state modulation of Kv channels in rat arterial SMC by cAMP-dependent PKA and Kv7.5 channel activity can influence RASMCs via cAMP/PKA activation [27, 28]. Allicin activated K_ATP_ channels in rat mesenteric arteries through PKA, and isoflurane activates PKA in rat VSMCs, which in turn activates K_ATP_ channels [29, 30]. Baicalin promoted relaxation of mesenteric by activation of BK_Ca_ through stimulation of the cAMP/PKA pathway [31]. Adenosine increased Kir currents via G protein-coupled receptor A3 through the activation of PKA in rabbit coronary arterial SMC [11]. PKA/cAMP may enhance VSMC phenotype switching. This research indicated that PDGF-BB increased Kir2.1 currents via PDGF-BBR*β* through the activation of PKA in RASMCs. However, Akt had no effects on Kir2.1 currents of PDGF-induced RASMCs. The single-channel research showed that AC increased NPo of Kir2.1 channel currents (Figure 5(d)).

PDGF-BB works by activating the PDGF-BBR*β* which activates AC, increasing cAMP and activating PKA. If this is the case, then applying PDGF-BB to inside-out patches should have no effect, yet Figure 1(e) suggests an immediate effect. The underlying mechanisms of the phenomenon maybe that PDGF-BB works by activating the PGDF-BB*β* receptor which activates AC, increasing cAMP and activating PKA, and activates the Kir2.1 channel in the other mechanism immediately.

The abnormal proliferation, migration, and phenotypic modulation of VSMCs are critical processes in atherosclerosis and restenosis [32, 33]. PDGF-BB can initiate a multitude of biological effects through the activation of intracellular signal transduction pathways that contribute to VSMC proliferation, migration, and phenotypic modulation [34]. Therefore, the inhibition of PDGF-induced VSMC proliferation, migration, and phenotypic modulation may represent an important point of therapeutic intervention in atherosclerosis and restenosis.

## 5. Conclusion

In conclusion, our study demonstrated that the activation of Kir2.1 channels by PDGF-BB results from the activation of the cAMP-PKA pathway via the PDGF-BB*β* receptor in RASMCs. Therefore, Kir2.1 may be a potential candidate for preventing or treating vascular diseases relevant to VSMC proliferation, migration, and phenotypic modulation.

## Data Availability

The datasets used and/or analyzed during the present study are available from the corresponding author on reasonable request.
